# Development of a multiplex qRT-PCR assay for the detection of porcine epidemic diarrhea virus, porcine transmissible gastroenteritis virus and porcine Deltacoronavirus

**DOI:** 10.3389/fvets.2023.1158585

**Published:** 2023-03-16

**Authors:** Yan Li, Jia-Wei Niu, Xia Zhou, Pin-Pin Chu, Kun-Li Zhang, Hong-Chao Gou, Dong-Xia Yang, Jian-Feng Zhang, Chun-Ling Li, Ming Liao, Shao-Lun Zhai

**Affiliations:** ^1^Guangdong Provincial Key Laboratory of Livestock Disease Prevention, Institute of Animal Health Guangdong Academy of Agricultural Sciences, Scientific Observation and Experiment Station of Veterinary Drugs and Diagnostic Techniques of Guangdong Province, Guangzhou, Guangdong, China; ^2^Maoming Branch Center of Guangdong Laboratory for Lingnan Modern Agricultural Science and Technology, Maoming, China

**Keywords:** porcine epidemic diarrhea virus, Transmissible Gastroenteritis Virus, Porcine Deltacoronavirus, porcine coronaviruses, multiplex real-time qRT-PCR

## Abstract

Currently, porcine coronaviruses are prevalent in pigs, and due to the outbreak of COVID-19, porcine coronaviruses have become a research hotspot. porcine epidemic diarrhea virus (PEDV), Transmissible Gastroenteritis Virus (TGEV), and Porcine Deltacoronavirus (PDCoV) mentioned in this study mainly cause diarrhea in pigs. These viruses cause significant economic losses and pose a potential public health threat. In this study, specific primers and probes were designed according to the *M* gene of PEDV, the *S* gene of TGEV, and the *M* gene of PDCoV, respectively, and TaqMan probe-based multiplex real-time quantitative reverse transcription-polymerase chain reaction (qRT-PCR) was developed for the simultaneous detection of PEDV, TGEV, and PDCoV. This method has high sensitivity and specificity, and the detection limit of each virus can reach 2.95 × 10^0^ copies/μl. An assay of 160 clinical samples from pigs with diarrhea showed that the positive rates of PEDV, TGEV, and PDCoV were 38.13, 1.88, and 5.00%; the coinfection rates of PEDV+TGEV, PEDV+PDCoV, TGEV+PDCoV, PEDV+TGEV+PDCoV were 1.25, 1.25, 0, 0.63%, respectively. The positive coincidence rates of the multiplex qRT-PCR and single-reaction qRT-PCR were 100%. This method is of great significance for clinical monitoring of the porcine enteric diarrhea virus and helps reduce the loss of the breeding industry and control the spread of the disease.

## Introduction

Coronaviruses belonging to the family Coronaviridae, the order Nidovirales, are single-stranded, positive-sense RNA viruses with the largest genome among known RNA viruses (1–3). Within swine enteric viruses, coronaviruses are the most devastating pathogens responsible for acute diarrhea, vomiting, dehydration, and high mortality in neonatal and suckling piglets (4–6).

According to the genetic and antigenic characteristics, all coronaviruses were divided into four genera: Alphacoronaviruses, Betacoronaviruses, Gammacoronaviruses, and Deltacoronaviruses (7). Novel coronavirus whole genome sequencing analysis showed that alpha-coronaviruses and beta-coronaviruses infected mammals; gamma-coronaviruses and delta-coronaviruses mainly infected birds, but some viruses could also infect mammals (6).

Coronaviruses, including PEDV, TGEV, and PDCoV, can cause diarrhea in piglets. PEDV and TGEV are alphacoronaviruses, while PDCoV is delta coronavirus.

PEDV was first isolated from porcine intestinal contents in the United Kingdom in 1978. Since then, PEDV has spread worldwide and isolated in many countries, including the USA, the UK, Argentina, Russia, and China, resulting in heavy economic losses to the porcine industry (8–10).

TGEV was first reported in 1946 in the United States, followed by outbreaks in many countries in the Americas, Asia, and Europe. TGEV has the greatest impact on piglets, especially those under 2 weeks of age, who are most susceptible to infection. Piglets often excrete feces containing undigested curds, with mortality rates often reaching 100% (11–13).

PDCoV was identified in pigs in Hong Kong in late 2012 (14). To date, PDCoV has been detected in at least 20 states in the United States, as well as in Canada, South Korea, China, Thailand, Lao People's Democratic Republic, Vietnam, and Mexico, posing a significant threat to the global swine industry (11, 15–19). In addition, PDCoV has been detected in poultry and humans, reflecting the potential for cross-species transmission (20, 21).

The clinical symptoms of the intestinal diseases caused by these swine coronaviruses are highly similar, and it can be challenging to distinguish them. It is important to note that coronaviruses tend to interspecies transmission, as exemplified by SARS-CoV-2 and PDCoV (22–27). By monitoring the epidemiology of coronaviruses in pigs, the potential for cross-species transmission of these viruses and the trend of cross-regional transmission of viruses can be well studied.

So far, PEDV, TGEV, and PDCoV have caused huge economic losses to the pig industry worldwide. In addition, some viruses' cross-species transmission ability may threaten public health. Therefore, developing a simple, rapid, accurate, and high-throughput detection method is necessary to distinguish porcine enteric coronaviruses. A multiplex qRT-PCR detection method based on TaqMan probes was established to detect three viruses, PEDV, TGEV, and PDCoV. This method will improve the virus's detection efficiency and accuracy while reducing the detection cost.

## Experimental section

### Primers and probes

To ensure the detection performance of the primers used in the multiplex qRT-PCR method, all available PEDV, TGEV, and PDCoV sequences were obtained and analyzed from GenBank. The primers and probes of PEDV, TGEV, and PDCoV were designed according to the *M* gene of PEDV, the *S* gene of TGEV, and the *M* gene of PDCoV. Three primer pairs and probes were designed using Oligo 6 (Version 6.44) software (Table 1). TaqMan probes for PEDV, TGEV, and PDCoV were labeled at the 5′-end with the reporter molecule: X-Rhodamine maleimide (ROX), pentamethylene cyanine (CY5) and 5(6)-carboxyfluorescein (FAM), respectively. The 3′-end of TaqMan probes were labeled with the quenchers: 8-Bromo-7-hydroxyquinoline 1 (BHQ1), 8-Bromo-7-hydroxyquinoline 2 (BHQ2), 8-Bromo-7-hydroxyquinoline 2 (BHQ2). Primers and probes were synthesized by Sangon Biotechnology (Shanghai) Co., Ltd. Primers were also used to construct plasmid standards.

**Table 1:** Primer and probe sequences.

**Target genes**	**Sequences (5^′^-3^′^)**	**Product size (bp)**
*PEDV-M*	F: ATCACYCTTATGCTGTGG ATAATGT	112
	R: CAGAAGTAGTGAGAAGCGCGT	
	Probe: Cy5-CGGTTGTGGCGCAGGACA CATT-BHQ1	
*TGEV-S*	F: TGAATGGCTCAATAGAA TTGAAAC	120
	R: CAACCTGTRCTACAACAGC AAAATAG	
	Probe: ROX-ATGGCCYTGGTATGTGT GGCTAC-BHQ2	
*PDCoV-M*	F: CACGCGTAAYCGTGTGATCTA	143
	R: CGGCAAAAVTTATGGACACA	
	Probe: FAM-TGGCTGCTCCAACCC TTCACCC-BHQ1	

The capital letters marked in red represent degenerate bases.

### Virus strains and field samples

RNA extraction and reverse transcription treat intestinal tissue or stool samples with 3 to 5 times PBS, vortex to mix, and collect supernatant after centrifugation at 12,000 × g for 15 min at 4°C. Nucleic acids were extracted using the E.Z.N.A.^®^ Total RNA Kit (Omega Bio-Tek, China) following the manufacturer's instructions. Reverse transcription was performed using the HiScript III RT SuperMix for qPCR (+gDNAwiper) kit (Nanjing Vazyme Biotechnology Co., Ltd.).

### Construction of plasmid standards

The target fragments of PEDV, TGEV, and PDCoV were amplified by PCR using PrimeScript™ High-Fidelity RT-PCR Kit [Bao Biomedical Technology (Beijing) Co., Ltd]. The PCR fragment was then cloned into a pUC57 vector [Takara Biomedical Technology (Beijing) Co., Ltd.] by TA colony and confirmed by DNA sequencing, p-PEDV-TGEV-PDCoV. The plasmid copy number was calculated, diluting the plasmids from 2.95 × 10^7^ to 2.95 × 10^0^ copies/μl. Single-reaction qRT-PCR was performed for each virus using the 10-fold diluted plasmids to generate standard curves.

### Reaction conditions of the single-plex qRT-PCR

As shown in Table 2, the total volume of the single-reaction qRT-PCR reaction was 25 μl. Amplification was carried out on Gentier 96R (Xi'an Tianlong Science and Technology) using the following program: 95°C 10 s; 40 cycles of 95°C 5 s, 60°C 30 s. The fluorescence signal was automatically collected at the end of each cycle, 35°C 30 s.

**Table 2:** Multiplex qRT-PCR reaction system.

**Reagent**	**Volume (μl)**
2 × One Step RT-PCR Buffer III	12.5
TaKaRa Ex Taq HS (5 U/ μl)	0.5
PrimeScript RT Enzyme Mix II	0.5
Primer-F (TGEV/PEDV/PDCoV)	0.8
Primer-R (TGEV/PEDV/PDCoV)	0.8
Probe (TGEV/PEDV/PDCoV)	0.3
Template	1
RNase Free dH_2_O	4.8
Total volume	25

### Optimization of the multiplex qRT-PCR

The multiplex reaction system was optimized using different volumes of primers (10 μM) and probes (10 μM). In the optimization stage, the number of primers added to the system was 0.4, 0.6, 0.8, 1.0, and 1.2 μl, respectively. The number of probes added to the system was 0.1, 0.2, 0.3, 0.4, 0.5, and 0.6 μl, respectively. The recombinant plasmid (2.95 × 10^7^ copies/μl) was used as the standard plasmid. Finally, each primer and probe concentration's fluorescence intensity and cycle threshold (Ct) value were compared. The same instrument and qRT-PCR program were used as described above. To obtain the best amplification efficiency, the annealing temperature was optimized. Twelve annealing temperature gradients were set up from 44 to 64°C, and each annealing temperature's fluorescence intensity and Ct values were compared.

### Sensitivity of the multiplex qRT-PCR assay

The standard plasmids were serially diluted 10-fold from 2.95 × 10^7^ to 0.295 × 10^0^ copies/μl (final concentration) for the standard plasmid and used as templates to evaluate the sensitivity of the developed assay, and the reaction does three repetitions at a time.

### Specificity of the multiplex qRT-PCR assay

To verify the specificity of the assay, positive samples for PEDV, TGEV, PDCoV, SADS-CoV, porcine rotavirus (PoRV), porcine pseudorabies virus (PRV), porcine circovirus 2 (PCV-2), porcine reproductive and respiratory syndrome (PRRSV) were used for detection by the developed multiplex qRT-PCR. All the samples were previously stored in our laboratory.

### Repeatability of the multiplex qRT-PCR assay

The assay was repeated three times with a 7-days interval, using 10-fold dilutions of the standard plasmid of each pathogen ranging from 2.95 × 10^7^ to 2.95 × 10^2^ copies/μl with three replicates per reaction. The coefficient of variation (CV) of the Ct values of the samples at each concentration in the three experiments was calculated to estimate repeatability.

### Clinical sample detection

To test the clinical application effect of the developed method, a total of 160 clinical samples collected from pigs with symptoms of diarrhea, including 70 small intestinal tissues and 90 anal swabs, were used for detection. At the same time, a single-plex qRT-PCR was used to verify the results, and the results were compared and analyzed.

## Results

### Establish a standard curve

**Figure 1:**
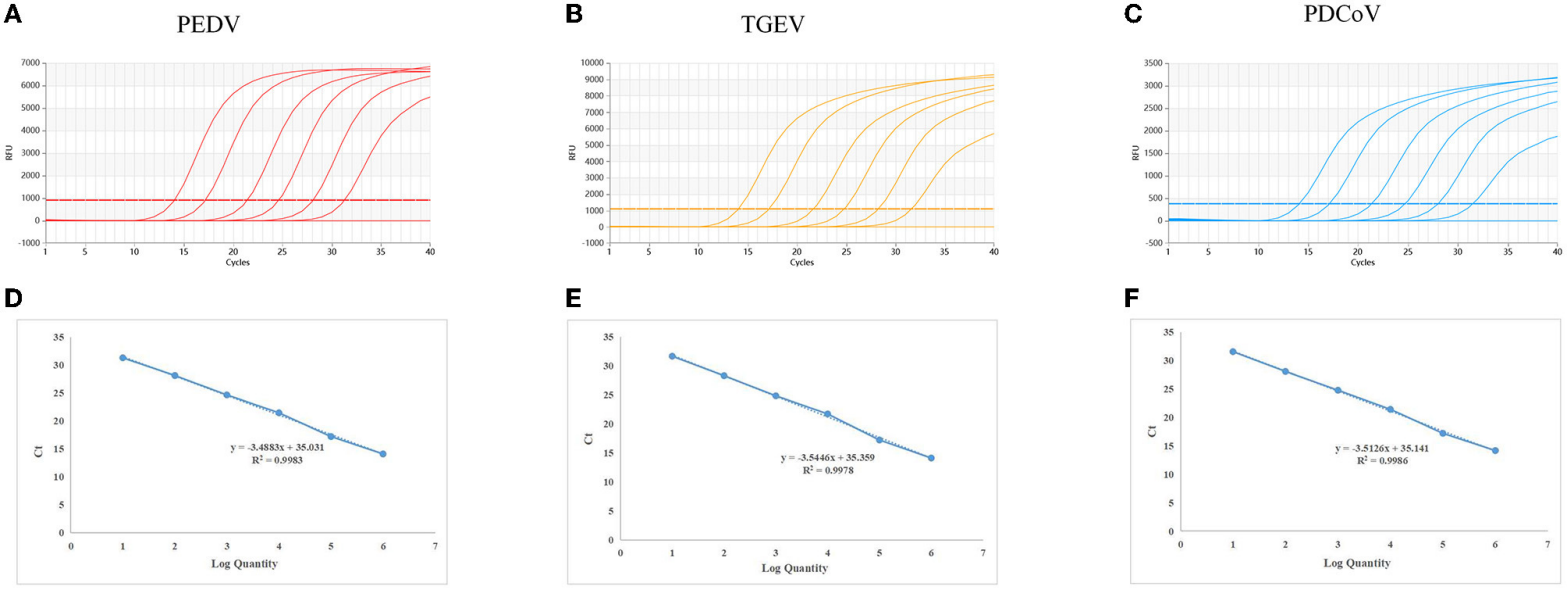
Dynamic curves and standard curves of the multiplex qRT-PCR: The dynamic curves were generated using the recombinant standard plasmids p-PEDV-TGEV-PDCoV for PEDV, TGEV, and PDCoV. The standard curves **(D–F)** were generated from the dynamic curves. In **(A–C)**, the plasmid concentrations of curves 1 to 6 ranged from 2.95 × 10^7^ to 2.95 × 10^2^ copies/μl; 7: Negative control.

The standard plasmids, ranging from 2.95 × 10^7^ to 2.95 × 10^2^ copies/μl, were used to create standard curves. The standard curves showed an acceptable amplification efficiency and correlation coefficient: PEDV, R^2^ = 0.9983; TGEV, R^2^ = 0.9986 and PDCoV, R^2^ = 0.9978, and these results showed that the designed primers and probes were effective (Figure 1).

### Optimization of the multiplex reaction system

The standard plasmids p-PEDV-TGEV-PDCoV carrying the target fragments were used as templates to optimize the reaction conditions of the multiplex qRT-PCR. The optimal annealing temperature and the concentrations of primers and probes were acquired based on orthogonal experiments.

**Figure 2:**
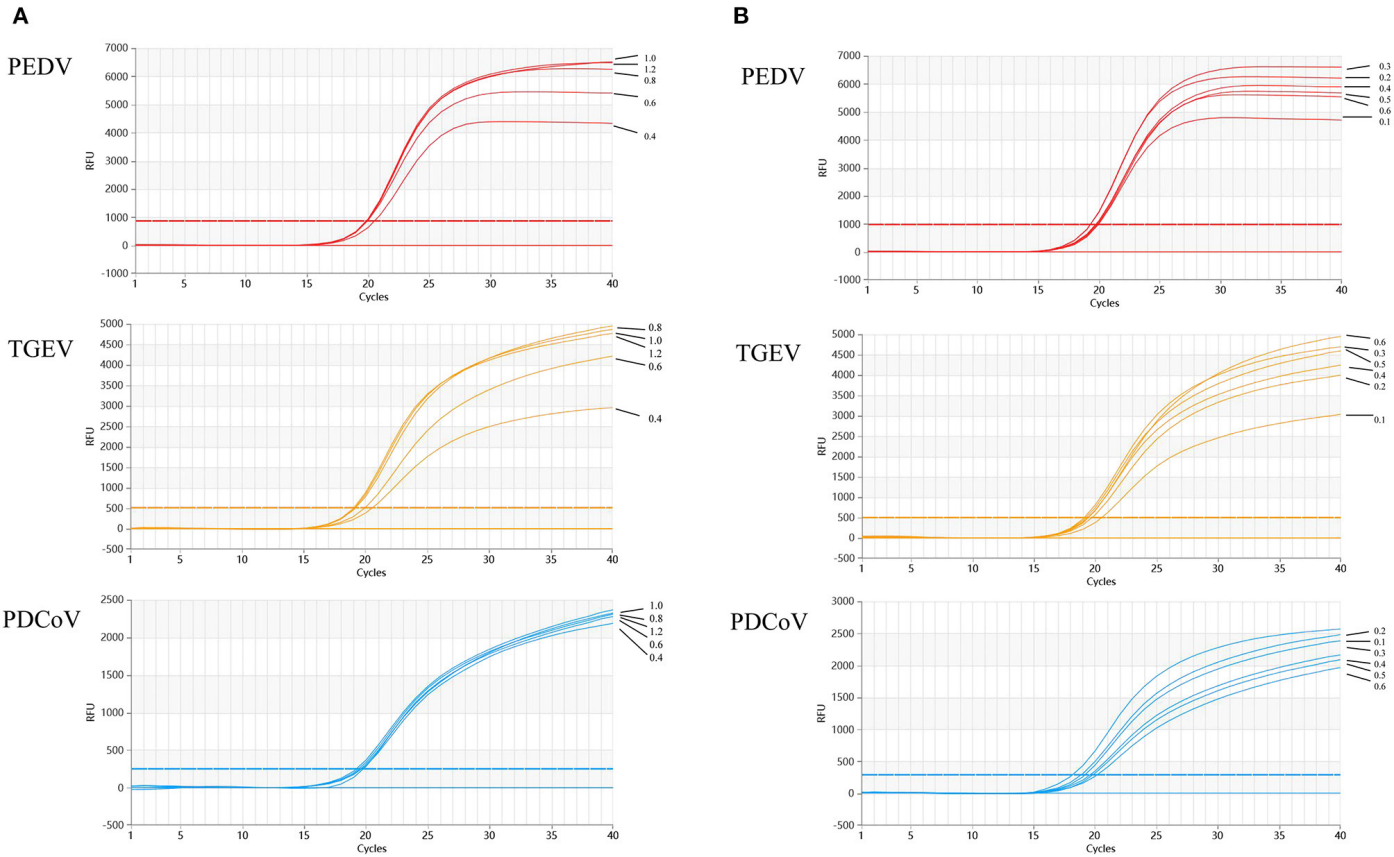
Optimization of multiplex qRT-PCR system. **(A)** shows the results of primers optimization in the reaction system, which showed that the optimal addition amount of upstream and downstream primers for TGEV and PEDV are 0.8 μl; the optimal addition amount of upstream and downstream primers for PDCoV is 0.6 μl. Optimization of multiplex qRT-PCR system. **(B)** Multiplex qRT-PCR probe volume optimizations. Probes optimization in the reaction system showed that the optimal addition number of probes for TGEV and PEDV is 0.3 μl; the optimal amount of probes for PDCoV is 0.2 μl.

**Figure 3:**
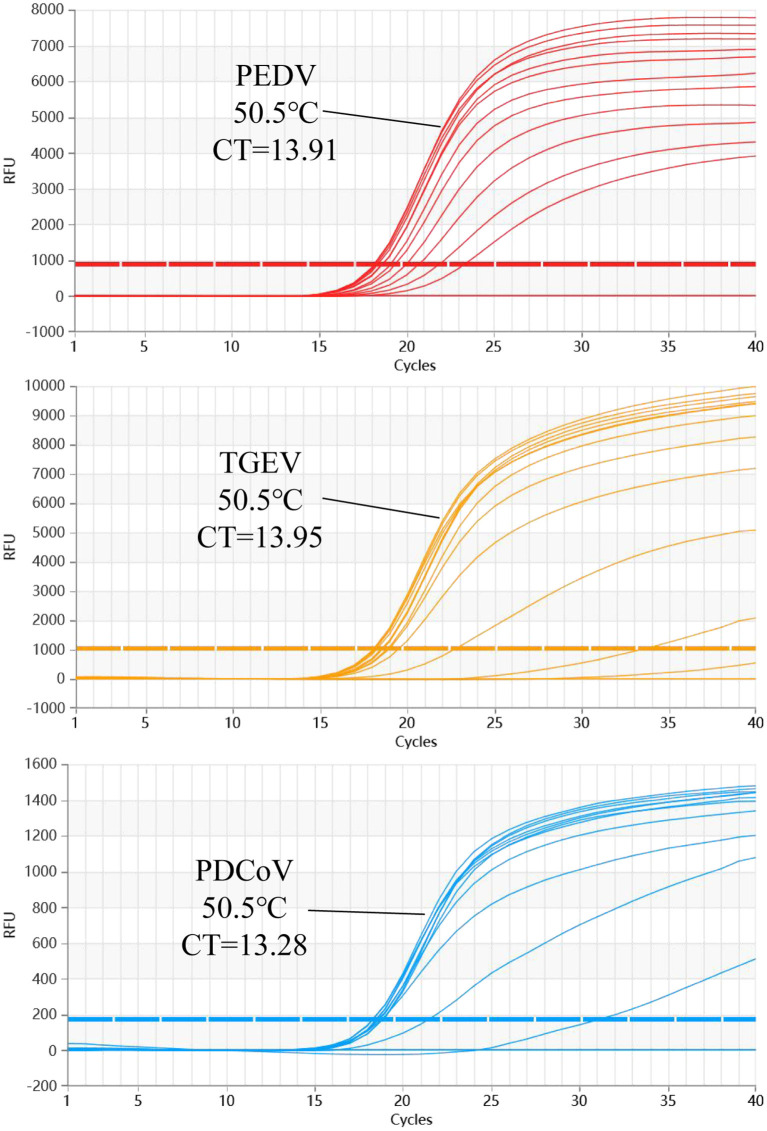
The multiplex qRT-PCR amplification curves of different Tm values. The annealing temperature optimization results of multiplex qRT-PCR. The results show that the amplification efficiency of this reaction is the highest when Tm = 50.5°C.

The results show that the optimal volume of probe and primer for PEDV and TGEV are 0.3 and 0.8 μl, respectively. The optimal volume of probes and primers for PDCoV were 0.2 and 0.6 μl, respectively (Figure 2). The results of annealing temperature optimization showed that 50.5°C was the optimal annealing temperature and the reaction had the best amplification efficiency (Figure 3).

### Sensitivity of the multiplex qRT-PCR assay

**Figure 4:**
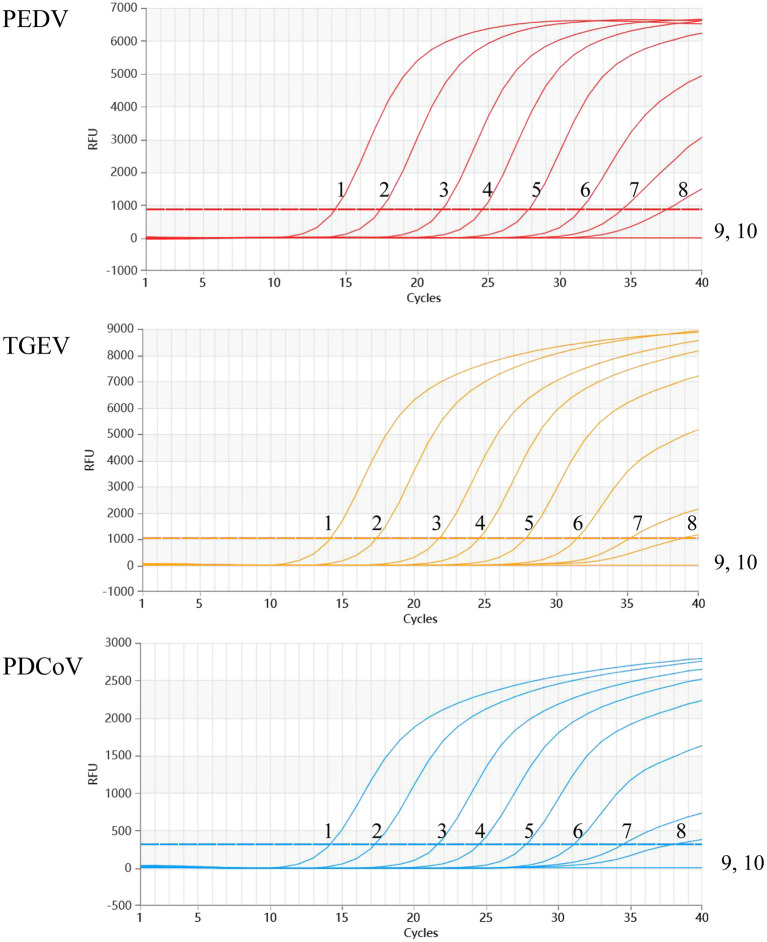
Sensitivity of the multiplex qRT-PCR assay. The dynamic curves were generated by using the recombinant standard plasmid p-PEDV-TGEV-PDCoV. 1-9: 2.95 × 107-0.219 × 100 copies/μl (final concentration); 10: Negative control.

The sensitivity results showed that the detection limit of the developed multiplex qRT-PCR assay was 2.95 × 10^0^ copies/μl of the standard plasmid (Figure 4).

### Specificity of the multiplex qRT-PCR assay

**Figure 5:**
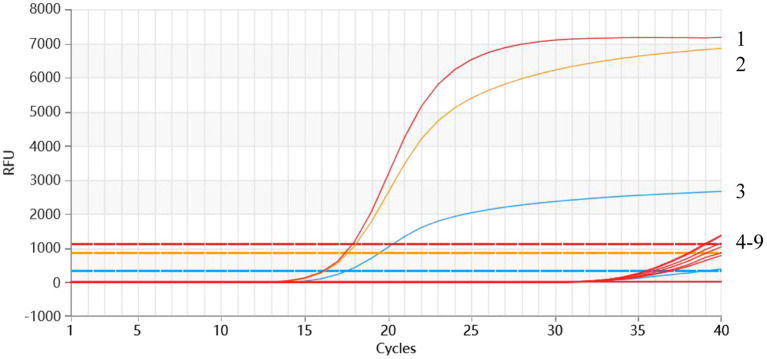
Specificity analysis of the multiplex qRT-PCR. 1-3: p-PEDV-TGEV-PDCoV (2.95 × 106 copies/μl); 4: PRRSV; 5: SADS; 6: PRV; 7: PoRV; 8: PCV2; 9: Negative control.

As shown in Figure 5, the positive samples for PEDV, TGEV, and PDCoV could be detected, while no amplification curve was observed with other viruses of pigs used in the assay.

### Repeatability of the multiplex qRT-PCR assay

The repeatability experiment was carried out with the serially diluted standard plasmids (2.95 × 10^7^ to 2.95 × 10^2^ copies/μl) as templates, and the Ct value of the experiment was calculated. As shown in Table 3, the coefficients of variation (CVs) of the intra- and inter-assay ranged from 0.12 to 1.22% and 0.38–1.34%, respectively, <2%. The experimental results showed that the developed multiplex method was stable.

**Table 3:** Repeatability results of the multiplex qRT-PCR assay.

**Plasmid**	**Concentration (copies/μl)**	**Ct values of intra-assay**			**Ct value of inter-assay**		
		$\bar{\text{x}}$	**SD**	**CV (%)**	$\bar{\text{x}}$	**SD**	**CV (%)**
PEDV	2.95 × 10^7^	14.25	0.05	0.36%	14.17	0.17	1.17%
	2.95 × 10^6^	17.52	0.02	0.12%	17.49	0.12	0.67%
	2.95 × 10^5^	21.96	0.25	1.14%	21.90	0.27	1.22%
	2.95 × 10^4^	24.77	0.11	0.45%	24.74	0.09	0.38%
	2.95 × 10^3^	28.03	0.17	0.59%	28.03	0.14	0.51%
	2.95 × 10^2^	31.49	0.19	0.62%	31.44	0.27	0.87%
TGEV	2.95 × 10^7^	14.22	0.04	0.25%	14.16	0.18	1.26%
	2.95 × 10^6^	17.49	0.02	0.09%	17.53	0.16	0.89%
	2.95 × 10^5^	22.02	0.23	1.04%	22.06	0.27	1.22%
	2.95 × 10^4^	24.80	0.12	0.46%	24.82	0.14	0.56%
	2.95 × 10^3^	28.10	0.16	0.56%	28.15	0.14	0.51%
	2.95 × 10^2^	31.45	0.12	0.37%	31.57	0.27	0.85%
PDCoV	2.95 × 10^7^	14.22	0.11	0.74%	14.06	0.19	1.33%
	2.95 × 10^6^	17.38	0.06	0.32%	17.34	0.08	0.45%
	2.95 × 10^5^	21.79	0.27	1.22%	21.77	0.29	1.34%
	2.95 × 10^4^	24.73	0.16	0.63%	24.67	0.20	0.79%
	2.95 × 10^3^	27.97	0.14	0.51%	27.93	0.16	0.57%
	2.95 × 10^2^	31.32	0.21	0.67%	31.28	0.32	1.01%

### Clinical sample detection

A total of 160 clinical samples, including 70 intestinal tissue samples and 90 anal swab samples from pig farms where diarrhea occurred in southern China, were tested by the developed multiplex qRT-PCR. The results of clinical sample tests showed that the positive rates of PEDV, TGEV, and PDCoV were 38.13% (61/160), 1.88% (3/160), and 5.00% (8/160), respectively. Additionally, the results also showed that 2 (1.25%) samples were coinfected by PEDV and PDCoV; 2 (1.25%) samples were coinfected by PEDV and TGEV; no coinfection of TGEV and PDCoV, and 1 (0.63%) sample was coinfected with PEDV, TGEV, and PDCoV. To verify the accuracy of the developed method, the clinical samples were detected by single-plex qRT-PCR, and the results of single-plex and multiplex qRT-PCR were compared and analyzed. The coincidence rate of the two methods was 100%, which confirmed that the results of the developed method were accurate and reliable (Table 4).

**Table 4:** Detection of clinical samples by the multiple and single-reaction qRT-PCR methods.

**Pathogens**	**Triple qRT-PCR**			**Single-reaction qRT-PCR**			**Coincidence rate**
	**Sample number**	**Positive**	**Percentage**	**Sample number**	**Positive**	**Percentage**	
PEDV	160	61	38.13	160	61	38.13	100%
TGEV	160	3	1.88	160	3	1.88	100%
PDCoV	160	8	5.00	160	8	5.00	100%
PEDV+ TGEV	160	2	1.25	160	2	1.25	100%
PEDV+ PDCoV	160	2	1.25	160	2	1.25	100%
TGEV+ PDCoV	160	0	0	160	0	0	100%
PEDV+ TGEV+ PDCoV	160	1	0.63	160	1	0.63	100%

## Discussion

In recent years, the viral diarrhea of piglets has still seriously threatened the development of the pig industry, causing major economic losses to global pig farmers. Clinically, PEDV, TGEV, and PDCoV are piglets' main pathogens causing viral diarrhea (28, 29). The epidemiological survey of pig diarrhea viruses showed that PEDV, TGEV, and PDCoV exhibited a trend of hybrid infection, including PEDV and PDCoV hybrid infections, PEDV and TGEV hybrid infections, and PEDV, TGEV, and PDCoV mixed infections (12, 30). The cause of the intestinal disease of piglets with hybrid infection of these viruses is becoming increasingly complicated. The clinical symptoms caused by PEDV, TGEV, and PDCoV exhibited high similarities, leading to the difficulty of determining the pathogens through clinical symptoms. Studies have shown that hybrid infections of multiple viruses may accelerate the common evolution of single and hybrid viruses or the reorganization of multiple viruses into new viruses, as reported by TGEV and PEDV reorganized strains in 2016 (31–34). Reorganization may produce intestinal virus strains or new viruses, which may cause potential outbreaks or popularity of pig viral diarrhea. Due to the global prevalence of SARS-CoV-2, coronavirus has attracted the great attention of scientists. It is worth noting that the newly discovered pork intestine coronavirus PDCoV has been detected in the infection cases of other species (including humans), indicating the great species crossing potential of PDCoV and a great threat to human public health (20).

Real-time quantitative fluorescent PCR is more sensitive, faster, and more accurate for detecting viral pathogens (35, 36). The multiplex RT-qPCR can detect and differentiate more than one pathogen in a single assay, which is particularly suitable for detecting mixed infection of multiple pathogens. In this study, a real-time multiplex PCR based on three pairs of specific primers and probes was developed for the differential detection of PEDV, TGEV, and PDCoV in one reaction. The developed multiplex qRT-PCR could specifically detect PEDV, TGEV, and PDCoV with the LOD of 2.95 × 10^0^ copies/μl for each pathogen. In multiplex qRT-PCR systems, the primers and probes must have good specificity. Because multiplex groups of oligonucleotides in the system will increase the possibility of non-specific amplification, it is necessary for probes and primers with excellent specificity.

The developed multiplex assay was also used to detect clinical diarrhea samples of piglets from southern China. Among the 160 samples, 61 samples were positive for PEDV, 3 samples for TGEV, and 8 samples for PDCoV, indicating that PEDV was still the major pathogen of piglet diarrhea, while the sporadic infections of PDCoV should also be highly concerned, as the increased detection rate of PDCoV in recent clinical samples. Furthermore, coinfection of PEDV, TGEV, and PDCoV had existed in certain pig herds, confirmed by the detection results that coinfection rates of PEDV/TGEV, PEDV/PDCoV, PEDV/TGEV/PDCoV were 1.25, 1.25, and 0.63%, respectively. Based on the results mentioned above, the developed multiplex real-time PCR could be a useful tool for rapid differentiation of PEDV, TGEV, and PDCoV in clinical samples from piglets with diarrhea and warning the infection of PDCoV.

## Conclusion

In conclusion, an excellent detection method should accurately reflect the pathogen's epidemiological data and help researchers monitor and prevent the virus more effectively. Although multiplex qRT-PCR has the advantages of saving time and capital costs, due to the high requirements for primers and probes, it still needs to be improved to develop effective multiplex qRT-PCR, often accompanied by many challenges. The development method can quickly and accurately detect three kinds of porcine diarrhea viruses, PEDV, TGEV, and PDCoV, simultaneously, effectively saving time and cost. The prevalence of different diarrhea viruses in piglets will increase the complexity of diarrhea and make it difficult to prevent and control. As a potential zoonotic pathogen, the prevalence of PDCoV in piglets should also attract continuous attention. Above all, more attention should be given to the molecular prevalence of PEDV, TGEV, and PDCoV, guiding precise prevention and control more effectively in the field.

## Data availability statement

The original contributions presented in the study are included in the article/supplementary material, further inquiries can be directed to the corresponding authors.

## Author contributions

YL, J-WN, and XZ performed the experiments and drafted the manuscript. J-WN, P-PC, and D-XY prepared materials for the experiments. YL and J-WN participated in the experiments. K-LZ, H-CG, C-LL, and S-LZ contributed to the data analysis. J-FZ, ML, and S-LZ conceived the study. All authors read and approved the final manuscript.
